# Nutritional status and growth performance of Fijian non-descript local chickens and their crosses with broilers under different production systems

**DOI:** 10.1007/s11250-024-03986-0

**Published:** 2024-07-10

**Authors:** Lorenzo T. Berukilukilu, Archibold G. Bakare, Paul A. Iji, Titus J. Zindove

**Affiliations:** 1 Department of Animal Science, School of Animal and Veterinary Sciences, Fiji National University, Nasinu, Fiji; 2 Faculty of Agriculture and Life Sciences, Lincoln University, Lincoln, 7647 Christchurch, New Zealand; 3 School of Environmental and Rural Sciences, University of New England, Armidale, Australia

**Keywords:** Crossbreeding, Scavenging system, Indoor production system, Nutrient intake, Sorghum, Kitchen waste

## Abstract

The study compared nutrient intake and growth performance of local chickens to that of local x broiler crossbreds under scavenging and indoor conventional systems. A total of 48 male and 48 female chickens for each of the two chicken types were allocated to four outdoor free-range pens. The chickens were allowed to scavenge whilst being supplemented with sorghum plus kitchen waste and broiler growers from week 5 to week 13 of age. The same design was repeated using the indoor conventional system. Local chickens and their crosses with broilers had higher growth rates under the scavenging system than the indoor production system (*P* < 0.05). Local chickens and their crosses with broilers had the same growth rates when fed the same diet (*P* > 0.05). Crop and gizzard contents from local chickens had the same crude protein as their crosses with broilers under both systems (*P* > 0.05). The crude protein values of crop and gizzard contents ranged from 25.4 to 30.4%. Crop and gizzard contents from scavenging chickens had energy content ranging from 16.2 to 17.1 MJ/Kg which was lower (*P* < 0.05) than that from chickens under the indoor conventional system (20.3 to 25.8 kJ/Kg). Iron content ranged from 655.7 to 1619.4 mg/Kg in scavenging chickens and 156.1 to 621.4 mg/Kg in enclosed chickens. Chickens of the same type had higher iron content in their crop and gizzard contents under the scavenging system than the conventional system (*P* < 0.05). Crossbreds between local chickens and broilers matches the scavenging abilities of the local chickens but have lower growth rates under the scavenging system.

## Introduction

Local chickens, which are commonly referred to as indigenous chickens, are regaining their popularity in many regions due to appreciation of their desirable traits such as adaptability to low-input scavenging production systems and highly desirable meat and eggs (Dessie et al. 2011). For example, local chickens in the Pacific region, Africa and Asia are known for producing tasty eggs and highly nutritious meat (Rashid et al. 2004; Zindove et al. 2022; Ranasinghe et al. 2024). Some of the reported meat characteristics of meat from local chickens include lower pH than broilers ranging from 5.5 to 6 (Rashid et al. 2004; Ranasinghe et al. 2024), low fat content ranging from 0.5 to 3% (Raphulu 2019; Ranasinghe et al. 2024) and crude protein ranging from 20 to 25% (Rajkumar et al. 2017; Raphulu 2019). Although high mature weights ranging from 1.2 to 2.5 kgs have been reported (Zindove et al. 2022; Ranasinghe et al. 2024), the local chickens are characterized by slow growth rates (Batkowska et al. 2015) which is one of the biggest concerns of most chicken producers.

Due to uncontrolled mating systems, conserving pure local chicken breeds or lines under the scavenging system in developing countries is a challenge (Dana 2011). As a result, local chicken populations in developing regions such as Africa, Asia and the Pacific Islands are mostly non-descript (Alders and Pym 2009; Zindove et al. 2022). In the quest of optimizing productivity under free range conditions, there has been a general drive towards the promotion of crossbreeding between local non-descript and fast-growing chickens such as broilers (Dana 2011). Although there is abundant literature on the characteristics of both local and improved chickens under different production systems (Batkowska et al. 2015; Castellini et al. 2016), their crossbreds are not well-characterized.

Local chickens and fast-growing improved breeds have distinguished nutritional status under different production systems due to their differences in scavenging abilities, digestive capabilities, and environmental requirements (Mekonnen et al. 2009). With most poultry productivity improvement programs promoting the use of crosses between local chickens and fast-growing improved breeds, it is important to determine how they perform under different production systems. Besides adaptability and growth performance, the nutritional status of chickens under any production system is of paramount importance. The type of nutrients that a chicken consumes, and their proportions determine growth performance, health status and the quality of its meat (Ferket and Gernat 2006).

For scavenging systems, determining the nutritional composition of crop and gut contents can be one of the ways to obtain information about nutrient intake of chickens (Raphulu and Jansen van Rensburg 2018; Yadav and Jha 2019). The chemical composition of crop and gizzard contents of slaughtered scavenging chickens indicate the nutritional composition of feed consumed by the chickens (Goromela et al. 2006). Previous studies have used nutrient composition of contents of the crops and gizzards separately (Prakash et al. 2020) and mixed (Ali 2002; Rahman et al. 2006) as indicators of nutrient intake by scavenging chickens. The nutrient intake of scavenging chickens depends on their scavenging ability and availability of supplements (Magothe et al. 2012). Commonly used supplements for the scavenging system include sorghum and kitchen waste (Etuk et al. 2012; Manuya et al. 2020). Although the scavenging ability of many local chicken breeds, as denoted by nutrient intake, has been extensively studied (Mwalusanya et al. 2002; Pousga et al. 2005; Raphulu 2019), such studies are limited for crossbreds between local chickens and fast-growing commercial breeds. Data on the performance of the crossbreds fed on common supplements such as sorghum and kitchen waste is also scanty.

In addition to human consumption, maize is widely used as a feed source for livestock production (Grote et al. 2021). As a result, there is high demand for maize worldwide which drives up its price. To reduce pressure on maize, programs aimed at improving the productivity and profitability of poultry farms explored the efficient use of alternative energy sources such as sorghum for enclosed production systems (Gualtieri and Rapaccini 1990; Alders and Pym 2009). Local chickens have been reported to efficiently digest whole sorghum under the scavenging system whilst sorghum-based diets are associated with inferior broiler performance under both scavenging and enclosed systems (Selle et al. 2018). To our knowledge, there is very little, if any, literature on the performance of crosses between broilers and local breeds supplemented with or fed on sorghum-based diets under any production system. Against this background, the objective of the study was to compare nutrient intake and growth performance of non-descript local chicken x broiler crossbreds to that of local chickens under different production systems. Such data will go a long way to evaluate the potential for genetic improvement of the productive efficiency of the local chickens through crossbreeding them with fast-growing improved breeds whilst maintaining their valuable properties such as good scavenging ability and utilization of lowly digestible feed sources. It was hypothesized that local chicken x broiler crossbreds’ crop and gizzard nutritional content and growth performance is higher than that of local chickens under both the scavenging and enclosed production systems.

## Materials and methods

### Study site

The study was conducted at the Fiji National University (FNU) crop farm in Koronivia. Koronivia is situated in Naitasiri district located at 18°0243.1 S and 178°3150.7 E and 11 m above sea level (Zindove et al. 2022). Naitasiri district is located on Fiji’s largest island, Viti Levu. It experiences a tropical climate characterized by rainfall throughout the year, totaling about 3500 mm (Zindove et al. 2022). The plots used for the experiment were previously used for planting cassava, maize, and taro. The plots currently have scattered coconut trees. There are also two contour ridges that are filled with still water throughout the year. The common grass species growing on the plots is Para grass (*Brachiaria mutica*). Ambient temperature and relative humidity were determined using data loggers. The average temperature and relative humidity for the experimental period were 27.3 ± 1.15 °C and 83 ± 2.25%, respectively.

### Experimental chickens

A total of 500 eggs from local scavenging chickens purchased from households in Ra, Nadroga-Navosa and Ba provinces were used to hatch chicks used in this experiment. The eggs were incubated until hatching at the FNU livestock farm. A total of 323 eggs hatched. A total of 500 local chicken x broiler breeder F1 crossbred day-old chicks were also purchased from a chicken farm in Ba province, Fiji and were reared at the FNU livestock farm. The crossbreds were obtained through crossbreeding non-descript chickens purchased from local communities with Cobb-500 broilers. The two batches of chicks were kept inside two separate brooders at 32ºC from day 1 to day 28 (4 weeks). The FNU livestock farm chicken house contains floors covered with about 5 cm thick layer of wood shavings and well-ventilated cages. A conventional starter diet and water were provided ad libitum until the chickens were four weeks old.

### Treatments and experimental design

Chickens were moved to the experimental site at the FNU crop farm at the start of week 5 of their age. They were sexed based on their phenotypic characteristics. Those with enlarged combs and wattles were confirmed as male (Tao and Walker 2000). From the total flock of each of the chicken types (local and local x broiler crossbreds), 96 males and 96 females were selected for use as experimental units. Stratified random sampling was used to select the chickens with sex as the stratum. The success rate of the sexing was confirmed to be 100% as the chickens grew older. The initial average individual bodyweight of the selected local and local x broiler crossbred chickens was 940 ± 11 g. The chickens were then allocated to 4 outdoor free-range pens and 4 indoor conventional system pens. For the outdoor free-range pens, two pens were allocated 12 cages each: 6 with male local chickens and the other 6 with male local x broiler crossbreds. The other two pens were also allocated 12 cages each: 6 with female local chickens and the other 6 with female local x broiler crossbreds. There were 4 chickens of the same sex and chicken type in each cage. Each cage represented an experimental unit. Two supplementary diets, sorghum + kitchen waste and broiler commercial grower diet, were then randomly allocated to pens with male chickens (one diet per pen). The same diets were also randomly allocated to the two pens with female chickens. The same design was repeated in the 4 pens in the indoor conventional system. Table 1 below illustrates the experimental design used. Each of the chickens was tagged using numbered chicken leg rings. Cages were also labeled with corresponding numbers of the allocated chickens. The free-range pens measured 900m^2^ each and were demarcated by a chain link fence reinforced by wooden poles. There was naturally growing mixed grass species dominated by Para grass (*Brachiaria mutica)*, crop residues and coconut waste inside the pens.

**Table 1:** Layout of the experimental design

	Scavenging system (Outdoor free-range pens)	Indoor conventional system pens
Sorghum + kitchen waste	**Males**	**Males**
	*Local chickens	
	*-6 cages, 4 chickens in each cage*	
	*Local x broiler Crossbreds	
	*- 6 cages, 4 chickens in each cage*	
Sorghum + kitchen waste	**Females**	**Females**
Broiler grower diet	**Males**	**Males**
Broiler grower diet	**Females**	**Males**

*Each cage represents an experimental unit*

^*^The same was repeated for the rest of the groups

The cages were wooden, measuring 2.5 × 2 × 1.5 m. The cages, with slatted floors, were elevated 1 m above the ground surface and fitted with wire mesh doors. For the scavenging system, the doors were left open during the day and the chickens were enclosed at night. A standard plastic drinker was placed near each cage to provide clean water. For the conventional production system, cages with the same design were used and placed in a chicken house. The chickens were enclosed throughout the experiment. All the experimental chickens were exposed to 12 h of natural light per day throughout the experiment.

Kitchen waste was collected from the university canteens and local restaurants at the end of every day during the experiment. It was mixed and ground using a NAIZEA electric dry–wet grinder. Sorghum grown at FNU crop farm was used as whole grain. For the scavenging system, 250 g of sorghum plus 250 g of ground kitchen waste were supplemented per cage every day for those on the sorghum plus kitchen waste diet. A total of 500 g of broiler grower diet were supplemented per cage for those on broiler grower diet. The amount of feed supplemented to the chickens was estimated based on supplementation rates by communal farmers reported in literature (Pousga 2007). Chickens in the conventional system were fed ad libitum as per experimental design.

### Growth performance

At the start of the experiment, all the chickens were weighed to determine the initial average weight. Body weight changes were determined by measuring the liveweight of chickens in each cage at the end of every week during the experiment. To determine the average weight of chickens in each cage, the chickens were put together in the crate and the weight of the crate with chickens was measured using a digital electronic scale. The weight of the crate was then subtracted from the total weight of the chickens and divided by four to get the average weight of chickens. The average daily gain (ADG g/bird/day) of the chickens was obtained by subtracting the initial average weight per cage from the final average weight per cage for each week and then dividing by 7 days.

### Slaughtering of chickens and sample collection

Eight weeks after the experiment commenced, all the chickens were slaughtered by cervical dislocation. The chickens were slaughtered between 1300hs and 1600 h. All the contents in crops and gizzards were collected and put into plastic bags within 20 min of slaughtering. The samples of the crop and gizzard contents were weighed and kept at 4ºC inside the refrigerator.

### Chemical analyses

To estimate nutrient intake by the chickens, crop and gizzard contents for chickens were sent for chemical analysis. Freeze-dried samples of the crop and gizzard contents were sent to the Fiji Agricultural Chemistry Laboratory at Koronivia Research Station for chemical analyses. Some of the samples from the crops of chickens were too small. Crop and gizzard samples were, therefore, mixed and analyzed together. Procedures described by AOAC (2005) were used to determine crude protein (CP), crude fat, calcium, sodium, iron, magnesium, manganese, copper, zinc and moisture content. Gross energy were analyzed using a bomb calorimeter (Par Instrument Co., Moline, IL) for crop and gizzard contents. All analyses were performed in duplicate.

### Statistical analyses

The effects of the production system, sex, feed regimen and chicken type on growth performance and chemical properties of the crop and gizzard contents were determined using the PROC GLM of SAS (2012). The following model was used: ${{\text{Y}}}_{{\text{ijklm}}}=\upmu +{{\text{P}}}_{{\text{i}}}+{{\text{C}}}_{{\text{j}}}+{{\text{F}}}_{{\text{k}}}+{{\text{S}}}_{{\text{l}}}+{\left({\text{CxP}}\right)}_{{\text{ij}}}+{\left({\text{FxC}}\right)}_{{\text{jk}}}+{{\text{E}}}_{{\text{ijklm}}}$

Where:

Y: average daily weight gain (AGD) and chemical properties of the crop and gizzard contents (moisture, crude protein, calcium, crude fat, phosphorus, magnesium, sodium, iron, manganese, copper and zinc levels in the crop and gizzard contents),
µ: overall mean,
P_i_: effect of i^th^ production system (indoor conventional system; scavenging system),
C_j_: effect of j^th^ chicken type (local chickens; local chickens x broiler crossbreds),
F_k_: effect of k^th^ feed regimen (sorghum + kitchen waste; broiler grower diet),
S_l_: effect of l^th^ sex (male, female),
C x P: interaction between production system and chicken type,
F x C: interaction between feed regimen and chicken type,
E_ijklm_: random error.

All first and second-order interactions were tested. Only significant interactions were included in the final model. Mean separation was performed using the LSMEANS using the PDIFF option (SAS 2012).

## Results

**Fig. 1:**
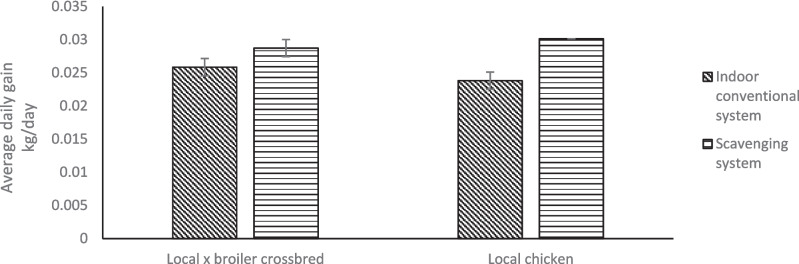
Average daily gain of local chicken and their cross with broilers under scavenging and indoor conventional systems

**Fig. 2:**
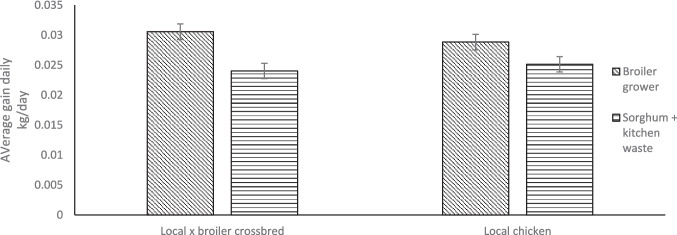
Average daily gain of local chickens and their cross with broilers fed on sorghum plus kitchen waste and broiler grower diet

**Fig. 3:**
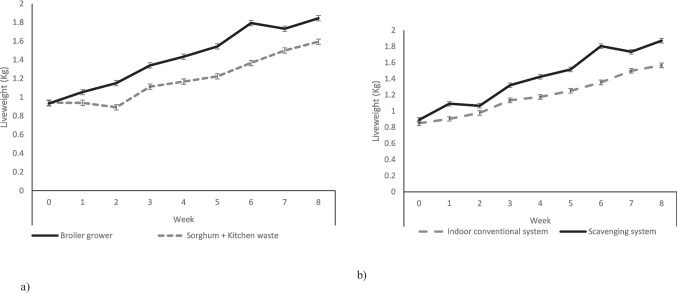
Liveweight **a**) local x broiler crossbred chickens fed on sorghum + kitchen waste and broiler grower diet over a period of 8 weeks **b**) local x broiler crossbred chickens under indoor conventional and scavenging systems

Figure 1 shows the growth performance of local chickens and their crosses with broilers under different production systems. Local chickens and their crosses with broilers had higher average daily weight gain under the scavenging system than the indoor production system (P < 0.05). Local chickens x broiler crossbreds had higher average daily weight gain than local chickens under the indoor conventional system and vice versa under the scavenging system (*P* < 0.05). Figure 2 shows the growth performance of local chickens and their crosses with broilers fed different feed regimens. For both chicken types, the average daily weight gain of chickens fed on broiler grower feed was higher than those on sorghum plus kitchen waste (*P* < 0.05). When on the same feed regimen, local chickens and their crosses with broilers had the same growth rates (*P* > 0.05). Irrespective of feed regimen and production system, the liveweight of local x broiler crossbred chickens increased after every 3 weeks (*P* < 0.05; Fig. 3a; Fig. 3b). Local x broiler crossbred chickens had higher liveweights from week 1 to 8 when fed on broiler growers than when fed on sorghum + kitchen waste diet (*P* < 0.05; Fig. 3a). Local x broiler crossbred chickens under the scavenging system had higher liveweights than those in the indoor conventional system from week 1 to week 8 (*P* < 0.05; Fig. 3b).

Table 2 shows the interaction of production system and chicken type on the nutrient and mineral composition of crop and gizzards contents. The moisture content of the crop and gizzard contents was lower under the indoor conventional system compared the scavenging system (*P* < 0.05). Under the scavenging system, local chickens had crop and gizzard contents with more moisture content than that from their crosses with broilers (*P* < 0.05) whilst there was no difference for those under the indoor conventional system (*P* > 0.05). The crop and gizzard contents from local chickens had the same crude protein and crude fat as their crosses with broilers under the scavenging system (*P* > 0.05). Local chickens under the indoor conventional system had the least crude fat content in crop and gizzard contents (*P* < 0.05). The energy content of crop and gizzard contents from scavenging chickens was lower than that from chickens under indoor conventional system (*P* < 0.05). Chickens of the same type had high iron content in their crop and gizzard contents under scavenging system than the conventional system (*P* < 0.05). Both Local chickens and their crosses with broilers had higher magnesium and sodium in crop and gizzard contents in the indoor conventional system than when scavenging (*P* < 0.05). Under the scavenging system, local chickens had lower manganese in crop and gizzard contents than their crossbreds with broilers (*P* < 0.05), but the zinc content was the same (*P* > 0.05).

**Table 2:** Interaction between production system and chicken type on nutrient and mineral composition of crop and gizzard contents

Nutrient composition	Scavenging system		Indoor conventional system	
	Local x broiler crossbred	Local chicken	Local x broiler crossbred	Local chicken
Moisture (%)	11.3 ± 0.67^a^	13.1 ± 0.77^b^	9.4 ± 1.04^c^	9.4 ± 0.50^c^
Crude protein (%)	30.1 ± 1.78^a^	30.4 ± 1.81^a^	28.7 ± 2.72^ab^	25.4 ± 2.87^b^
Crude fat (%)	12.5 ± 1.18^a^	17.4 ± 5.98^a^	16.5 ± 5. 39^a^	10.3 ± 0.59^b^
Gross energy (MJ/kg DM)	17.1 ± 0.55^c^	16.2 ± 1.47^c^	20.3 ± 0.78^b^	25.8 ± 2.63^a^
Calcium (%)	0.8 ± 0.13	0.8 ± 0.19	1.3 ± 0.37	1.0 ± 0.37
Phosphorus (%)	0.9 ± 0.05	0.9 ± 0.06	1.1 ± 0.20	1.1 ± 0.17
Potassium (%)	1.3 ± 0.09	1.3 ± 0.15	1.2 ± 0.13	1.4 ± 0.14
Magnesium (%)	0.2 ± 0.02^b^	0.2 ± 0.03^b^	0.3 ± 0.02^a^	0.3 ± 0.03^a^
Sodium (%)	0.5 ± 0.02^c^	0.5 ± 0.04^c^	1.4 ± 0.65^a^	0.6 ± 0.05^b^
Iron (mg/kg)	1619.38 ± 353.75^a^	655.7 ± 155.25^b^	621.4 ± 160.40^b^	156.1 ± 38.01^c^
Manganese (mg/kg DM)	178.6 ± 19.71^a^	120.3 ± 28.83^b^	178 ± 43.49^ab^	127.8 ± 43.69^ab^
Copper (mg/kg DM)	45 ± 7.38	49 ± 12.63	87 ± 42.26	99.5 ± 68.54
Zinc (mg/kg DM)	323.3 ± 59.52^a^	280.2 ± 62.80^ab^	275.3 ± 55.24^ab^	218.1 ± 42.05^b^

^ab^ Means with different superscripts, within a row, are different (*P* < 0.05)

Table 3 shows the interaction of feed regimen and chicken type on the nutrient composition of crop and gizzard contents. Local chickens fed sorghum plus kitchen waste had the highest energy content in their crop and gizzard contents when compared to local chickens fed on broiler grower diet and crossbreds fed on both feed regimens (*P* < 0.05). Irrespective of chicken type, chickens fed on sorghum and kitchen waste had lower Zn, and Ca levels in their crop and gizzard contents than those fed on broiler grower diets (*P* < 0.05). Local chickens x broiler crossbreds chickens fed on sorghum and kitchen waste had lower P levels in their crop and gizzard contents than chickens fed on broiler grower diets (*P* < 0.05). Local chickens fed on sorghum and kitchen waste had lower Cu levels in their crop and gizzard contents than the rest of the chickens (*P* < 0.05).

**Table 3:** Interaction between feed regimen and chicken type on nutrient and mineral composition of the crop and gizzard contents

Nutrient composition	Sorghum plus kitchen waste		Broiler grower diet	
	Local x broiler crossbred	Local chicken	Local x broiler crossbred	Local chicken
Moisture (%)	10.0 ± 0.99^b^	10.4 ± 0.87^ab^	10.8 ± 0.86^ab^	11.9 ± 0.77^a^
Crude protein (%)	28.8 ± 2.53	27.5 ± 2.40	29.9 ± 2.05	29.4 ± 2.88
Crude fat (%)	11.5 ± 1.55	14.5 ± 4.69	17.5 ± 6.75	11.6 ± 0.64
Gross energy (MJ/kg DM)	18.9 ± 0.92^b^	27.3 ± 4.32^a^	18.5 ± 0.89^b^	19.1 ± 1.81^b^
Calcium (%)	0.7 ± 0.22^b^	0.4 ± 0.13^b^	1.4 ± 0.28^a^	1.4 ± 0.26^a^
Phosphorus (%)	0.8 ± 0.05^c^	0.9 ± 0.08^bc^	1.3 ± 0.15^a^	1.1 ± 0.15^ab^
Potassium (%)	1.1 ± 0.10^b^	1.5 ± 0.14^a^	1.3 ± 0.11^ab^	1.2 ± 0.09^b^
Magnesium (%)	0.3 ± 0.02^a^	0.2 ± 0.03^b^	0.2 ± 0.03^b^	0.2 ± 0.03^b^
Sodium (%)	0.7 ± 0.16	0.6 ± 0.06	1.2 ± 0.66	0.6 ± 0.02
Iron (mg/kg DM)	1297.6 ± 406.22^a^	558.3 ± 182.13^bc^	943.1 ± 219.13^ab^	420.3 ± 156.42^c^
Manganese (mg/kg DM)	143.4 ± 42.16^b^	52.8 ± 16.15^c^	213.3 ± 12.38^a^	194.5 ± 26.69^ab^
Copper (mg/kg DM)	74.8 ± 43.12^a^	24.4 ± 4.92^b^	57.3 ± 9.25^a^	121.5 ± 65.59^a^
Zinc (mg/kg DM)	168.8 ± 18.76^c^	155.1 ± 34.02^c^	429.8 ± 39.26^a^	334.6 ± 36.97^b^

^ab^ Means with different superscripts, within a row, are different (*P* < 0.05)

## Discussion

There is increasing interest in combining the hardiness of local chickens with the feed efficiency of fast-growing improved breeds through crossbreeding. As such, it is important to understand the performance of the crossbreds between local and fast-growing improved chickens under different production systems. The finding that both local chickens and their crossbreds with broilers had higher growth rates and liveweights under the scavenging systems contradicts literature. Li et al. (2017) found that the growth performance of medium-growing chickens was higher under cage and indoor-floor systems than the free-range system. Li et al. (2011) also found that the performance of Chinese local chickens under scavenging systems was lower than under indoor systems. Limited feed resource base, exposure heat and increased exercise whilst scavenging have been attributed as the major causes of poor growth performance of scavenging chickens (Ncobela and Chimonyo 2016). The observed differences might be due to differences in shed provision in form of coconut trees in the pens, supplementation and a rich feed resources base found under tropical conditions. Although the growth rate of local chickens x broiler crossbreds was higher under the scavenging system than the indoor conventional system, the chickens can still be regarded as slow growing because they took 3 weeks to have significant gain in liveweight. Studies to ascertain the influence of shade on performance of scavenging chickens are necessary.

The observed growth performance differences between local chickens and their crossbreds with broilers under the two production systems are more likely to be genetic. Youssao et al. (2012) suggested that differences in growth performance of chickens under the same production system can be attributed to differences in their genetic make-up. At can, therefore, be insinuated that local chickens in this study grow better than their crosses with broilers under the scavenging system because they are more suited to the system whilst the crosses do better than local chickens under indoor systems. The finding that local chickens and their crosses with broilers had the same growth rates when fed on the same feed regimen was unexpected. Khawaja et al. (2012), Castellini et al. (2016) and Mancinelli et al. (2023) reported that crossbreds between fast-growing and hardy slow-growing chickens have better growth performance than their purebred counterparts. The unexpected findings warrant further exploration. The current study used sorghum plus kitchen waste as the only supplements for the scavenging system. Under most chicken scavenging systems, there is a wide range of supplements such as maize, millet and sunflower (Dana 2011; Mekonnen et al. 2009). More studies on the performance of crossbreds between local chickens and fast-growing commercial breeds with a wide range of supplements such as maize, sunflower and millet are necessary.

The observed differences in moisture content in gizzard and crop contents of scavenging and non-scavenging chickens can be due to differences in diet, exercise, and environmental conditions. In tropical climates, scavenging chickens consume more water than those indoors because they are exposed to high temperatures and exercise more as they scavenge (Abioja and Abiona 2021). Scavenging chickens also consume feed resources such as fresh plant leaves and grass which have high water content (Ncobela and Chimonyo 2015). The high moisture content can therefore be ascribed to the high water intake of scavenging chickens coupled with low water loss due to humid conditions. High moisture content in the gizzard and crop contents may promote digestive activities in the gastrointestinal tract of the chickens (Rodrigues and Choct 2018). Scavenging chickens should, therefore, be constantly supplied with drinking water especially under hot conditions. Studies to compare the actual water intake of the chickens under the different productions systems are necessary.

The finding that local chickens had gizzard and crop contents with more moisture content than their crosses with broilers might be an indication of genotype-based physiological differences among scavenging chickens as an adaption to the production systems. Before making concrete conclusions, further studies on the physiological and behavioral factors behind the observed differences in moisture content of the gizzard and crop contents are necessary. Once the genotype-based physiological differences are ascertained, it would be recommended that, under the scavenging system, crossbreds between local chickens and broilers should be constantly provided with cool clean water for proper physiological performance.

High fat content in gizzard and crop contents of scavenging chickens resonated with Pousga et al. (2005) and Mwalusanya et al. (2002) who argued that fat intake by scavenging chickens can be high in tropical areas due to abundance of fat sources such as insects and worms among others for the scavenging chickens. The same reason can be tributed to the high iron content in gizzard and crop contents for scavenging chickens than those kept indoors. Low energy content in gizzard and crop contents of scavenging chickens as compared to those under indoor systems might be an indication that the scavenging feed resource base had low energy sources. Energy has been reported as a major limiting nutrient for scavenging chickens (Rashid et al. 2004; Ncobela and Chimonyo 2016). Fijian farmers with scavenging chickens should, therefore, be encouraged to supplement their chickens with energy sources. There is need to explore on affordable non-conventional energy sources which can be used to supplement scavenging chickens in Fiji.

The energy, fat and trace elements intake of scavenging chickens depend on the scavenging ability of the chickens (Ncobela and Chimonyo 2016). The fact that crosses between local chickens and broilers herein had gizzard and crop contents with the same energy, zinc, sodium, magnesium and fat content as that from local chickens under the scavenging system shows that they have similar scavenging abilities for trace elements, energy, and fat sources. In agreement to findings herein, Raphulu et al. (2015) reported low concentrations of trace elements in crop contents of scavenging local chickens. The finding that, under the scavenging system, crosses between local chickens and broilers had more Mn and iron than local chickens could be an indication that they ingest more soil which is the main source for the two trace elements for scavenging chickens (Raphulu et al. 2015). Basing on the findings herein, Fijian farmers keeping crosses between local chickens and broilers under scavenging systems do not need to supplement with zinc, sodium, magnesium sources. The scavenging resource base, however, differs with geographical location within Fiji (Zindove et al. 2022). It is, therefore, necessary to replicate the study in different geographical locations within Fiji before making blanket recommendations on supplementing scavenging local chickens and broilers crossbreds with sources of trace elements.

The finding that energy content in gizzard and crop contents of local chickens fed sorghum plus kitchen waste was higher than broilers x local chickens crossbreds on the same diet could because of the variability in composition of kitchen waste and, thus nutrient content. One of the challenges for scavenging chicken systems is the large daily and seasonal variability in nutritional content of feed sources such as kitchen waste (Alshelmani et al. 2021). The feed sources for scavenging systems can, sometimes, have higher energy content than conventional feed sources (Raphulu 2019) as observed in this study were gizzard and crop contents from local chickens fed on kitchen waste plus sorghum diet had more energy content than those fed on broiler diets. This is, however, not consistent, and the ability to adapt the variability in nutrient content of the feed sources is a desirable characteristic for scavenging chickens.

The finding that trace elements such as Zn, Ca and P were low in gizzard and crop contents of chickens fed on kitchen waste and sorghum than those fed on broiler growers’ diet was expected. Although sorghum grain is a rich source of minerals including magnesium zinc and calcium (Mabelebele et al. 2015), a sorghum plus kitchen waste only diet cannot meet the chickens’ Cu, Zn, Ca and P requirements as it cannot match the levels in broiler growers’ diet. This might also be part of the explanation of why chickens fed on growers had higher growth rates and liveweights than those on sorghum plus kitchen waste. Irrespective of genotype, feeding non-scavenging chickens with sorghum plus kitchen waste only is, therefore, not recommended.

## Conclusion

Crosses between local chickens and broilers have similar scavenging abilities as denoted by the same energy and fat content in their crop and gizzard contents. The crosses between local chickens and broilers had high levels of trace elements in their crop and gizzard contents further indicating their scavenging abilities. Indoor chickens fed on kitchen waste plus sorghum diet have low levels of trace elements such as Cu, Zn, Ca, and P. Although crosses between local chickens and broilers grew better than local chickens under indoor systems but not under scavenging systems, their growth performance was better under scavenging systems than indoor systems. It can be concluded that crossbreds between local chickens and broilers match the scavenging abilities of the local chickens but not their growth performance under scavenging systems. Further studies on feed intake and nutrient digestion of the crossbreds between local chickens and broilers are necessary. The study was conducted using flocks which were scavenging on station. It’s necessary to replicate the study using village flocks in different geographical locations. Considering that the feed resource base for scavenging chickens vary with season, it is also important to evaluate seasonal variation in performance scavenging local chickens x broiler crossbreds. Future studies should also investigate the nutritional composition of gizzard and crop contents of scavenging local chickens x broiler crossbreds separately.

## Data Availability

Data will be made available on request.
